# Rezum Therapy for Benign Prostatic Hyperplasia: Dubai’s Initial Experience

**DOI:** 10.7759/cureus.18083

**Published:** 2021-09-18

**Authors:** Rashed Rowaiee, Aya Akhras, Jeyaseelan Lakshmanan, Zuhair Sikafi, Farhad Janahi

**Affiliations:** 1 Urology, Mohammed Bin Rashid University of Health and Medical Sciences, Dubai, ARE; 2 Epidemiology and Biostatistics, Mohammed Bin Rashid University of Health and Medical Sciences, Dubai, ARE; 3 Urology, Mediclinic City Hospital, Dubai, ARE

**Keywords:** benign prostatic hyperplasia, transurethral radiofrequency thermal ablation, water vapor therapy, lower urinary tract symptoms, minimally invasive

## Abstract

Introduction

Symptomatic benign prostatic hyperplasia (BPH) is a condition that affects middle-aged men, leading to a decreased quality of life secondary to symptoms of difficult urination, urinary frequency, urgency and nocturia. The treatment modalities of this pathology include pharmacologic and invasive interventions, both of which vary in effectiveness and they come with a myriad of side effects. Recent advancements have allowed for the development of Rezum, a minimally invasive and effective approach to treating BPH while maintaining a good safety profile with comparable outcomes to other treatment modalities.

Methods

We retrospectively identified 49 patients with symptomatic BPH who underwent Rezum therapy in one center in Dubai, the United Arab Emirates between January and December 2020. We assessed several parameters related to their condition including prostate volume, pre-operative and post-operative post-void residual (PVR) and peak urinary flow (Qmax) number of treatments given, trial without catheter and mean date of follow-up. Safety and side effects were also assessed.

Result

Our sample included symptomatic men with a mean age of 64 (10) who had a mean follow-up time of three months (IQR 2-5.2), median prostatic volume of 58 cc (IQR 44-82) and a mean of (SD 33.9). The initial Qmax and PVR were 7.3 ml/s (IQR 5.5-10.3) and 80.4 cm^3 ^(IQR 43.4-120.0) respectively, and post-operative Qmax and PVR were 16.3 ml/s (SD 5.7) and 20.7 cm^3^ (IQR 16.2-28.2). Post-operatively, we observed a significant increase in Qmax of 8.11 ml/s (p=0.001) post-operatively, a mean decrease of 94.32 cm^3^ (p=0.001) in PVR. The favorable outcome parameters in Qmax and PVR demonstrate the efficacy of this procedure. We have also noted that the change in Qmax and PVR does not vary by initial prostate volume.

Conclusion

In this report, we aimed to highlight the benefit, efficacy and safety of offering Rezum to patients with symptomatic BPH in a single center in Dubai, reporting on the first United Arab Emirates experience with this novel procedure. This procedure confers the benefit of being minimally invasive, safe and effective, and with lower rates of sexual dysfunction compared to medical therapy or trans-urethral resection of the prostate (TURP). It is associated with similar outcomes to TURP, and an improvement in quality of life, while carrying a low-risk profile. Our experience corresponds with the available literature regarding the efficacy and satisfaction rates of Rezum for BPH patients. We hope that results from this study encourage further investigation into the long-term implications of Rezum, up to five years post-operatively.

## Introduction

Lower urinary tract symptoms (LUTS) are very common and distressing urological symptoms encountered among middle-aged men [1]. In 40% of men over 50, these symptoms are attributable to benign prostatic hyperplasia (BPH) [2], a non-malignant hyperplasia of the periurethral zone of the prostate, resulting in either irritative or obstructive symptoms which significantly impair their quality of life.

Over the years, many treatments have been devised to treat BPH. Initial medical therapy may be effective for mild to moderate symptoms. However, in patients with moderate to severe symptoms, with objective measurements indicating a greater degree obstruction surgical intervention may be necessary. Previously, trans-urethral resection of the prostate (TURP) was the most performed procedure, considered the gold standard for treating BPH. However, this resulted in high levels of morbidity including sexual dysfunction, urethral strictures, stress incontinence, bleeding and a longer length of stay [3,4]. More recently, many innovative surgical interventions have been introduced using thermal energy and water vapor, mechanical expansion with UroLift, as well as prostatic artery embolization [5,6]. All of these are aimed to avoid the complications associated with TURP while maintaining comparable outcomes.

 Rezum is indicated for men ≥ 50 years of age with BPH and prostate volumes ranging from 30 cm^3^ to 80 cm^3^. It is also indicated for the treatment of BPH of the central zone and/or a median lobe involvement. Overall, the prostate is ablated via convective water vapor thermal energy, generated via radiofrequency [7,8]. This procedure has been reported in the literature to result in a significant reduction in LUTS in patients with BPH, along with an appreciable safety profile [9]. Rezum has also demonstrated improvement in symptoms scores compared to medical therapy [10,11]. Another key distinguishing benefit of Rezum therapy is low rates of sexual dysfunction post-operatively, which is a key complication observed in other operative procedures for BPH, such as TURP [10].

Results of this study will be crucial in influencing the choice of care for patients regarding this novel procedure with consideration of the prostate sizes presented in the Arab world, to optimize and better facilitate their healthcare experience.

## Materials and methods

The Rezum procedure uses convective water vapor to deliver thermal energy to prostatic tissue in 9-second bursts. In our facility, patients underwent Rezum treatment in the operating theatre under general anesthesia. All patients were inserted a two-way Foley catheter of variable sizes post-operatively.

Data collection and cohort selection

We retrospectively queried the Health Information System (HIS) for patients who underwent the Rezum procedure since its introduction in January 2020 to December 2020 at Mediclinic City Hospital, Dubai, United Arab Emirates with a diagnosis of BPH using the ICD9 code 600.01. The variables collected included basic demographic information (age and ethnicity) as well as pre-operative and post-operative values, such as prostatic volume, prostate-specific antigen (PSA) levels, post-void residual (PVR) and maximum flow rate (Qmax). In addition, we collected data on number of treatments given, time to removal of catheter (TWOC), average time to follow up and complications. Standardized symptom questionnaire scoring was unobtainable due to a lack of compliance by patients. We also further subclassified the patients into two groups based on prostate volume: prostate volume <80cc and prostate volume >80cc, to compare the efficacy of the procedure in prostate volumes larger than the recommended cut-off by the Rezum System Guidelines.

Statistical methods

Data were entered and analyzed using IBM Statistical Package for the Social Sciences (SPSS) software version 25.0 (IBM Corp., Armonk, NY). The change (pre/post) in outcomes such as PSA, Qmax and PVR were computed. As the change was negatively skewed and that the observations are paired, Wilcoxon signed-rank test was used. The p-value of 0.05 was considered for statistical significance. Additionally, we sought to investigate whether the initial prostatic volume was related to the change in Qmax and PVR, using a non-parametric Mann-Whitney U test.

## Results

A total of 49 patients, from 13 different countries undergoing Rezum surgery at Mediclinic City hospital between January and December 2020 were identified. The mean age was 64 years (SD 10) (Table 1). Overall, 73.4% (36/49) of patients had prostate gland volumes larger than 80cc. The median prostatic volume was 58 cc (IQR 44,82) with a mean of 68 cc (SD 33.9). The mean number of treatments was 4.9 treatments into the median lobe, with a maximum number of 10 treatments. Mean duration of post-operative follow-up was found to be three months (IQR 2,5.2).

**Table 1:** General demographical data. Qmax: peak urinary flow; PSA: prostate-specific antigen; PVR: post-void residual; TWOC: time to removal of catheter.

	Mean/median	SD/IQR
Age	64	10
Prostate volume	58	44, 82
Initial PSA total	3	1,6
Initial Qmax	7.3	5.5, 10.3
Initial PVR	80.4	43.4, 120.0
TWOC	5	3,7
Post-Op Qmax	16.3	5.7
Post-Op PVR	20.7	16.2, 28.2

**Figure 1:**
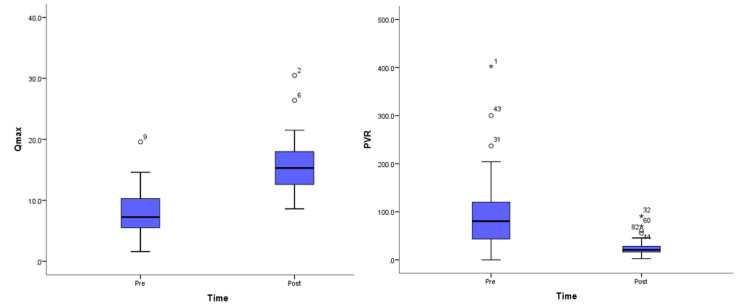
Change in Qmax and PVR pre- and post-operatively. Qmax: peak urinary flow; PVR: post-void residual.

Our sample had a mean initial total PSA of 3 (IQR 1,6). Mean initial Qmax of 7.3 ml/s (IQR 5.5, 10.3), initial PVR 80.4 cm^3^ (IQR 43.4, 120), mean days until catheter removal post-operative was five days (IQR 3,7). The mean post-operative Qmax was 16.3 ml/s (SD 5.7) and post-operative PVR 20.7 cm^3 ^(IQR 16.2, 28.2). Qmax levels increased significantly, with an average of 8.11 ml/s (SD 7.42) (p<0.001). Similarly, significant reductions in PVR were noted by an average of 94.32 cm^3^ (SD 94.68) (p<0.001) (Table 2; Figure 1). We observed no significant changes in Qmax or PVR among prostates < 80cc versus prostates >80cc (p-value: 0.488, 0.424, respectively) (Table 3).

**Table 2:** Mean changes in Qmax and PVR. Qmax: peak urinary flow; PVR: post-void residual.

	Mean	SD	Median	Percentile 25	Percentile 75	p-value
Change in Qmax	8.11	7.42	5.50	3.50	12.40	0.000
Change in PVR	-94.32	94.68	-71.80	-146.00	-30.20	0.000

**Table 3:** Mean changes in objective measures of Qmax and PVR in relation to prostate volume. Qmax: peak urinary flow; PVR: post-void residual.

		Mean	SD	Median	Percentile 25	Percentile 75	p-value
Change in Qmax							
Prostate volume	<=80	7.20	5.96	6.50	2.90	12.40	0.488
	>80	12.37	13.30	5.50	3.90	27.70	
Change in PVR							
Prostate volume	<=80	28.1	24.6	20.5	14.7	27.9	0.424
	>80	29.9	20.5	26.4	16.6	45.5	

Due to the diversity of our sample, spanning patients from 13 different countries, we sought to investigate a correlation between larger prostate volume and different ethnicities (Arab versus Non-Arab). Overall, 71.4% (35/49) of the population was Arab, of those, 28.6% (10/35) had a prostate size greater than 80 cc. Among the Non-Arab group, only 21.4% (3/14) had prostate sizes greater than 80 cc. The correlation between prostate size and ethnicity was non-significant (p-value: 0.609).

The complications observed were mild, including one case of urinary tract infection (UTI) which was treated with oral antibiotics and two cases of hematuria which spontaneously resolved. Some patients reported mild discomfort due to the presence of the catheter temporarily post-operatively.

No sexual dysfunction was reported by any of the patients who had undergone this procedure. Regarding the use of post-operative medications, patients stopped using their medications within three months of the procedure.

## Discussion

The spectrum of interventions available for treating BPH has expanded in recent years. The introduction of Rezum posed as a novel surgical innovation, offering satisfactory clinical outcomes while maintaining a safe and minimal side effect profile [3]. It has been recommended by the American Urological Association [AUA] and the European Urological Association (EUA) alongside the existing interventions of watchful waiting and lifestyle changes, pharmacotherapy and surgical management for the treatment of LUTS. It has been historically challenging for BPH patients to remain compliant to the medical treatments offered, which include 5-α reductase and phosphodiesterase inhibitors, which offer symptomatic relief at the expense of side-effects that threaten compliance.

In this study, we have highlighted the effectiveness of Rezum therapy through the improvement of pre-operative and post-operative outcomes in a single institution in Dubai, among an ethnically diverse population, including prostate volumes greater than 80cc. Our study showed no significant difference in Qmax or PVR among prostate volumes of less than 80cc versus greater than 80cc. Additionally, a correlation between ethnicity and prostate sizes was non-significant.

The gold standard of treating BPH historically has been TURP, which provided patients with clinical improvement regarding their LUTS. The major drawback of TURP is attributed to its complications, most notably bleeding and sexual dysfunction [12]. Rezum has the advantage of improving clinical outcomes while preserving sexual function, and minimal bleeding. Furthermore, this procedure can be done without general anesthesia, which may be favorable for some older patients. It has also been evaluated for cost-effectiveness in the USA when compared to long-term follow-up with TURP, which showed Rezum to be comparative in health and financial burden [13,14]. The literature has indicated that while clinical improvement was increased with TURP, due to lower adverse effects with Rezum, an overall reduction in cost is observed [10,14] [15]. Additionally, randomized control trials have shown a reduction in symptomatic LUTS (storage and voiding symptoms) at four years with a mean IPSS improvement of 47% with Rezum therapy [10]. Lastly, due to the COVID-19 pandemic, and the favorability of reducing operative times, Rezum proves as a suitable option with each procedure reported to average around [17.5 minutes] when compared to 60-90 minutes with TURP [16].

Since the introduction of Rezum, there has been little data on the efficacy of this novel therapy in the Arab population. The United Kingdom has reported on its initial experience as described by Maximilian et al, which showed results similar to those we have found [17]. In our study, we have seen the benefit conferred by Rezum therapy among the Arab population, as evidenced by improvements in Qmax and PVR, as well as patient symptom reporting. Our population had no patients who were catheter dependent, of which 14 (28.5%) were on medical treatment. Within 90 days post-operatively, our patients had stopped their previous medications; this is in accordance with Mollengarden et al’s single office experience [18].

In our study, we focused on the changes in PVR and Qmax as objective reference points of improvement in post-operative outcomes. We observed a significant mean increase in Qmax at three months follow-up, as well as a significant reduction in PVR. These numbers corroborate other internationally published papers [17,19].

We also investigated whether patient’s initial prostatic volume was related to changes in PVR and Qmax, which showed no statistically significant correlation in our sample. This is in contrast with Garden et al. which showed that men with larger prostates (>80cc) demonstrated more profound changes in Qmax and PVR than in those men with smaller prostates (<80cc) [17].

Patients on medical treatment for BPH suffer from medication side-effects, leading to non-compliance; as demonstrated by Cindolo et al. who showed that adherence after one year with at least six months of therapy was 29% in a population-based cohort study of 1.5 million men [20]. In our experience, patients only required medical treatment temporarily post-operatively, following which no medications were required for their symptom control at 90 days post-operatively. We believe that this factor alone may increase the acceptability of the procedure and would lead to higher adoption rates.

In addition to a decreased need for medications, and increased quality of life, Rezum is a procedure with a good safety profile. Temporary catheterization post-operatively is one of its major drawbacks. This lasted an average of five days in our experience, during which our patients reported discomfort. In our experience, the complications observed included UTI, which was managed by antibiotics only, and two cases of spontaneously resolving hematuria. No patients required readmission for any reason. Furthermore, no patients reported on sexual dysfunction, up to the latest follow-up, this is in line with published data. Dixon et al. reported that no clinically significant changes in sexual function were observed over two years. Additionally, McVary et al. reported that a single treatment water vapor therapy has no negative impact on sexual function over a three-year period in contrast with medical treatment which lead to worsening erectile function and libido [9,10].

We hypothesized that Arab men were more likely to suffer from larger prostate volumes compared to non-Arabs. Our study findings did not show any significant association between ethnicity and prostate volumes. Further studies among larger samples are needed to confirm this.

Lastly, the population requiring surgical intervention for BPH entails an older male population, many of which may be on anticoagulation and suffering from multiple co-morbidities. Rezum poses a great option as there is no need to interrupt anticoagulation and avoids the need for general anesthesia.

Limitations

This study is not without limitations. Firstly, our study undertook a retrospective design, which is prone to selection bias that may underestimate negative outcomes. We have not been able to report on IPSS or AUASS due to the lack of participation by patients in our sample, this impacts the objectivity of our data. We have therefore used measures such as Qmax and PVR changes to qualify for the objectivity of our dataset.

Since the introduction of Rezum in January 2020, we had anticipated to conduct this study on a larger cohort; however, due to the emergence of COVID-19 and the halting of elective surgical procedures, this was not possible. Overall, this has led to a smaller sample size than anticipated, which affects the power of our study. Moreover, the rates of retreatment, and post-operative variables [PSA, prostate volume] may be underreported due to the cancellation of planned subsequent procedures by the pandemic, and patient hesitancy to visit the hospital, respectively.

Moreover, some patients were lost to follow-up due to living in a different Emirate and to transport difficulties, limiting follow-up data. Furthermore, there is variability in patient follow-up, many of whom have incomplete or missing data. There were insufficient measurements of prostatic median lobe size to sufficiently analyze its impact on outcomes and responsiveness to Rezum. Our small sample size contains patients with prostate sizes of >80cc, and highlights the need for future larger, more comprehensive prospective studies to further elucidate Rezum outcomes for patients with large prostates.

## Conclusions

From our study, we conclude that Rezum water vapor thermal therapy is a safe, effective and minimally invasive surgical option for the treatment of men with moderate to severe LUTS. This procedure has demonstrated efficacy among different ethnicities and larger prostate volumes. We hope that the results of our study encourage further long-term studies into the efficacy of Rezum among different patient populations, as well as its long-term efficacy.

## References

[1] Wei JT, Calhoun E, Jacobsen SJ (2005). Urologic diseases in America project: benign prostatic hyperplasia. J Urol.

[2] Zhang SJ, Qian HN, Zhao Y (2013). Relationship between age and prostate size. Asian J Androl.

[3] Foster HE, Dahm P, Kohler TS, Lerner LB, Parsons JK, Wilt TJ, McVary KT (2019). Surgical management of lower urinary tract symptoms attributed to benign prostatic hyperplasia: AUA Guideline Amendment 2019. J Urol.

[4] Guo RQ, Yu W, Meng YS (2017). Correlation of benign prostatic obstruction-related complications with clinical outcomes in patients after transurethral resection of the prostate. Kaohsiung J Med Sci.

[5] Jones P, Rai BP, Nair R, Somani BK (2015). Current status of prostate artery embolization for lower urinary tract symptoms: review of world literature. Urology.

[6] Jones P, Rajkumar GN, Rai BP, Aboumarzouk OM, Cleaveland P, Srirangam SJ, Somani BK (2016). Medium-term outcomes of urolift (minimum 12 months follow-up): evidence from a systematic review. Urology.

[7] Green Z, Westwood J, Somani BK (2019). What's new in rezum: a transurethral water vapour therapy for BPH. Curr Urol Rep.

[8] Mynderse LA, Hanson D, Robb RA (2015). Rezūm System Water Vapor Treatment for Lower Urinary Tract Symptoms/Benign Prostatic Hyperplasia: Validation of Convective Thermal Energy Transfer and Characterization With Magnetic Resonance Imaging and 3-Dimensional Renderings. Urology.

[9] Dixon CM, Cedano ER, Pacik D (2016). Two-year results after convective radiofrequency water vapor thermal therapy of symptomatic benign prostatic hyperplasia. Res Rep Urol.

[10] McVary KT, Rogers T, Roehrborn CG (2019). Rezūm water vapor thermal therapy for lower urinary tract symptoms associated with benign prostatic hyperplasia: 4-year results from randomized controlled study. Urology.

[11] Gupta N, Rogers T, Holland B, Helo S, Dynda D, McVary KT (2018). Three-year treatment outcomes of water vapor thermal therapy compared to doxazosin, finasteride and combination drug therapy in men with benign prostatic hyperplasia: cohort data from the MTOPS trial. J Urol.

[12] Ahyai SA, Gilling P, Kaplan SA (2010). Meta-analysis of functional outcomes and complications following transurethral procedures for lower urinary tract symptoms resulting from benign prostatic enlargement. Eur Urol.

[13] Arezki A, Sadri I, Couture F (2021). Reasons to go for Rezūm steam therapy: an effective and durable outpatient minimally invasive procedure. World J Urol.

[14] Ulchaker JC, Martinson MS (2018). Cost-effectiveness analysis of six therapies for the treatment of lower urinary tract symptoms due to benign prostatic hyperplasia. Clinicoecon Outcomes Res.

[15] Darson MF, Alexander EE, Schiffman ZJ (2017). Procedural techniques and multicenter postmarket experience using minimally invasive convective radiofrequency thermal therapy with Rezūm system for treatment of lower urinary tract symptoms due to benign prostatic hyperplasia. Res Rep Urol.

[16] Johnston M, Shah T, Emara A (2019). Rezūm water vapour ablation therapy for benign prostatic hyperplasia: initial results from the United Kingdom. J Urol.

[17] Johnston MJ, Noureldin M, Abdelmotagly Y (2020). Rezum water vapour therapy: promising early outcomes from the first UK series. BJU Int.

[18] Mollengarden D, Goldberg K, Wong D, Roehrborn C (2018). Convective radiofrequency water vapor thermal therapy for benign prostatic hyperplasia: a single office experience. Prostate Cancer Prostatic Dis.

[19] Garden EB, Shukla D, Ravivarapu KT, Kaplan SA, Reddy AK, Small AC, Palese MA (2021). Rezum therapy for patients with large prostates (≥ 80 g): initial clinical experience and postoperative outcomes. World J Urol.

[20] Cindolo L, Pirozzi L, Fanizza C (2015). Drug adherence and clinical outcomes for patients under pharmacological therapy for lower urinary tract symptoms related to benign prostatic hyperplasia: population-based cohort study. Eur Urol.

